# Effects of low frequency electric fields on synaptic integration in hippocampal CA1 pyramidal neurons: implications for power line emissions

**DOI:** 10.3389/fncel.2014.00310

**Published:** 2014-10-09

**Authors:** Francesco Cavarretta, Nicholas T. Carnevale, Domenico Tegolo, Michele Migliore

**Affiliations:** ^1^Institute of Biophysics, National Research CouncilPalermo, Italy; ^2^Department of Mathematics and Informatics, University of PalermoPalermo, Italy; ^3^Department of Neurobiology, Yale University School of MedicineNew Haven, CT, USA

**Keywords:** hippocampal CA1 neuron, realistic model, simulation, extracellular field

## Abstract

The possible cognitive effects of low frequency external electric fields (EFs), such as those generated by power lines, are poorly understood. Their functional consequences for mechanisms at the single neuron level are very difficult to study and identify experimentally, especially *in vivo*. The major open problem is that experimental investigations on humans have given inconsistent or contradictory results, making it difficult to estimate the possible effects of external low frequency electric fields on cognitive functions. Here we investigate this issue with realistic models of hippocampal CA1 pyramidal neurons. Our findings suggest how and why EFs, with environmentally observed frequencies and intensities far lower than what is required for direct neural activation, can perturb dendritic signal processing and somatic firing of neurons that are crucially involved in cognitive tasks such as learning and memory. These results show that individual neuronal morphology, ion channel dendritic distribution, and alignment with the electric field are major determinants of overall effects, and provide a physiologically plausible explanation of why experimental findings can appear to be small and difficult to reproduce, yet deserve serious consideration.

## Introduction

Electric transmission lines and household appliances are ubiquitous, and there is an increasing awareness of the possible influence on biological and cognitive processes that can be related to the electromagnetic fields they generate (see for example the EMF Rapid Program, 2002). Experimental investigations on humans have given inconsistent or contradictory results (reviewed in Crasson, 2003). For example, acute effects of exposure to electric fields have not been found in linesmen working with high-voltage power lines (Gamberale, 1989), and a meta-analysis revealed little consistent evidence that fields have any effect on cognitive function (Barth et al., 2010), but psychological and mental health variables of humans living near transmission lines have shown significant differences related to exposure (Beale et al., 1997). These findings suggest that there might be some (not well understood) physiological conditions at the cellular and brain circuits level that can generate this kind of extreme variability and confused experimental findings at the behavioral and psychological level. A number of computational studies have been published on the effects of external electric fields (EFs) on neurons. For example, it has been suggested that a stationary and uniform EF can robustly alter the balance between theta and gamma rhythms in a network of CA1 neurons (Berzhanskaya et al., 2013), and that a stationary but non-uniform EF can differentially modulate the spatial distribution of dendritic membrane potential of a morphologically detailed passive neuron (Anastassiou et al., 2010). It has also been suggested (Radman et al., 2007) that subthreshold oscillating fields can perturb spike timing.

For many years we have used computational models closely linked to experimental observations of the anatomical and biophysical properties of hippocampal neurons to study electrical signals generated by neuronal activity (e.g., Migliore, 2003; Gasparini et al., 2004; Ferrante et al., 2008; Shah et al., 2011; Miceli et al., 2013). We hypothesized that this empirically-based approach might lead to new insights about the effects of electrical fields on neuronal excitability and synaptic integration. In this paper we address the effects of EFs, uniform or oscillating at power line frequency, on biophysically and morphologically detailed models of hippocampal pyramidal CA1 neurons. The rationale for this choice is that there is ample experimental evidence (reviewed, for example, in Squire et al., 2007) and theoretical support (reviewed in Morris, 2006) for the paramount role of the hippocampus on cognitive tasks. This is especially true for pyramidal neurons in the CA1 region, because of their critical position as the main output stage of hippocampal circuitry (Johnston and Amaral, 2003).

Existing exposure guidelines (IEEE, 2002; ICNIRP, 2010) were designed to prevent direct excitation of action potentials in neurons and synaptic terminals. Since we were interested in the possible cognitive effects, field strengths in this study were limited to subthreshold intensities. We investigated the functional consequences, and the possible importance for cognitive processes, of exposure to EFs, and how such consequences may be affected by individual neuronal morphology, ion channel dendritic distribution, and orientation relative to the field. The results suggest a physiological explanation for inconsistent experimental findings, and provide new data and experimentally testable predictions regarding neural effects of power line emissions.

## Materials and methods

Simple models consisting of one or two cylinders were employed to address the elementary effects of EFs on neuronal structures. To study the interaction of fields with detailed neuronal architecture and biophysically accurate membrane properties, we used two full 3D reconstructions of hippocampal CA1 pyramidal neurons taken from previous works [cell 5038804 from Migliore et al. (2005) ModelDB entry 55035, and cell c62564 from Migliore et al. (2008), ModelDB entry 87535]. In all cases, the same standard, uniform, passive properties were used, with values τ_m_ = 28 ms and *R*_a_ = 150 Ω·cm for the membrane time constant and cytoplasmic resistivity, respectively. For simulations involving active properties, the set and distribution of sodium, *DR*- and *A*-type potassium conductances, and *h*-current (*Na*, *K_DR_*, *K_A_*, and *I_h_*, respectively), were identical to those used in previous works and already validated against several experimental findings in CA1 neurons (e.g., Migliore, 2003; Marcelin et al., 2009; Ascoli et al., 2010). Briefly, the *Na* and *K_DR_* were uniformly distributed over the entire neuron, whereas *K_A_* and *I_h_* increased linearly with distance from the soma. The EF was modeled as described in Berzhanskaya et al. (2013), with additional custom code to control field direction and frequency modulation. A schematic representation of the EF implementation is shown in Supplementary Figure S1.

Excitatory synaptic inputs (with a reversal potential of 0 mV) were randomly distributed on the proximal (*n* = 150) and distal (*n* = 50) dendrites, taking into account experimental findings (Megías et al., 2001), and modeled with a double-exponential conductance change with a reversal potential of 0 mV and rise and decay time constants of 2 and 10 ms, respectively. Unless stated otherwise, the same peak conductance was used for all synapses in any given neuron (0.2 *nS* for cell 5038804 and 0.045 *nS* for cell c62564). These values resulted in approximately the same average firing frequency (25.4 Hz and 22.5 Hz for cell 5038804 and cell c62564, respectively) during random (poisson) stimulation at 50 Hz in the absence of an EF, allowing a direct comparison of the results between the two neurons.

Except for a few specific cases noted in the Results Section, simulations intended to probe perturbations of neuronal function that may occur *in vivo* near power lines assumed a frequency of 50 Hz and a tissue field intensity of 40 V/m; this value is based on the relative dielectric constant of 67.8 for gray matter (Voigt et al., 2011) and environmental measurements around power line pillars that found local field amplitudes up to 2520 V/m (Anderle et al., 1996). Simulations designed to explore the relationship between neuronal properties and EF effects used field intensities that were chosen so as to produce clear results; this is consistent with the common experimental practice of using “physiologically unrealistic” manipulations of ionic concentrations, pharmacological treatments, applications of channel blockers etc. as needed to reveal or isolate a particular phenomenon of interest.

All simulations were implemented with v7.3 of the NEURON simulation environment (Hines and Carnevale, 1997) on desktop PCs. We have made the model and simulation files used in this work available for public download under the ModelDB section of the Senselab database http://senselab.med.yale.edu (Migliore et al., 2003) under accession number 151731.

## Results

**Figure 1:**
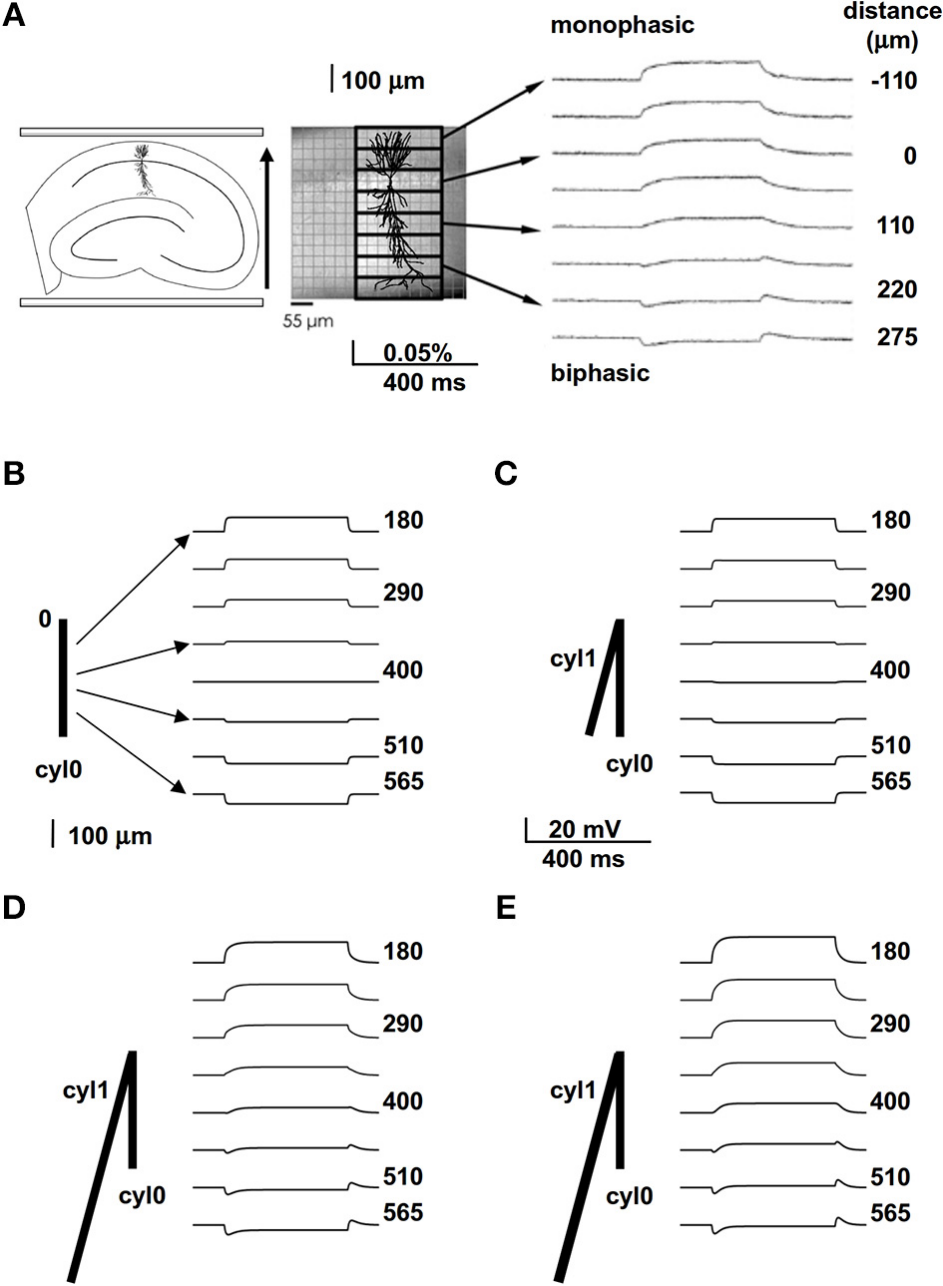
**A spatially extended morphology takes into account the biphasic membrane polarization in the presence of an external field**. **(A)** Experimental (*in vitro*) preparation (*left* and *middle*) and findings (*right*) showing the membrane polarization of hippocampal pyramidal CA1 neurons at different distances from the soma (taken and adapted from Figure 10 of Bikson et al., 2004); the arrow on the left indicates the field orientation. **(B)** A passive cylinder (length 800 μm) was subjected to a uniform 40 V/m electric field, aligned with its length, that turned on and off abruptly. The traces show membrane potential observed at different distances from site “0”; note the absence of any biphasic polarization. **(C)** Adding cylinder cyl1, which is identical to cyl0 but oriented at an angle of 30° relative to the field, results in a very small biphasic polarization that is most noticeable at 400 μm; **(D,E)** increasing the length from 800 to 3200 μm **(D)** or the diameter from 1 to 1.5 μm **(E)** of cyl1 results in biphasic polarizations similar to those observed experimentally. In all panels the field orientation was as in **(A)**.

We started with simplified models designed to provide a qualitative understanding of how neuronal properties might account for EF effects that others have observed *in vitro*. For example, Bikson et al. (2004) used a pair of parallel plates to apply a uniform EF of −40 V/m aligned with the major apical trunks of CA1 pyramidal neurons in hippocampal slice. They stained the whole slice with a potentiometric dye (RH414, Molecular Probes, Eugene, OR, USA), so that they could observe perturbations of membrane potential by optical recording at the “tissue level” of resolution. They found that the EF effect on dendritic membrane potential depended on the relative position and orientation of the dendrites with respect to the field (Figure 1A, modified from Figure 10A of Bikson et al., 2004). Dendrites at different spatial locations were polarized in qualitatively different ways, with dendrites close to the soma showing monophasic responses to field onset and offset (Figure 1A, right, upper traces), while distal dendrites displayed a biphasic course with a transient peak followed by a sag when the EF was turned on and off (Figure 1A, right, lower traces). Biphasic responses were observed in distal dendritic regions, independent of field direction (Bikson et al., 2004).

This phenomenon is interesting, since this region-dependent excitatory/inhibitory action of the EF could interfere with dendritic signal integration, and thus with cognitive processes. It has been hypothesized (Omori et al., 2009) that non-uniform membrane resistance *R*_m_—specifically a drastic decrease of *R*_m_ with distance from the soma—could be responsible for this effect.

**Figure 2:**
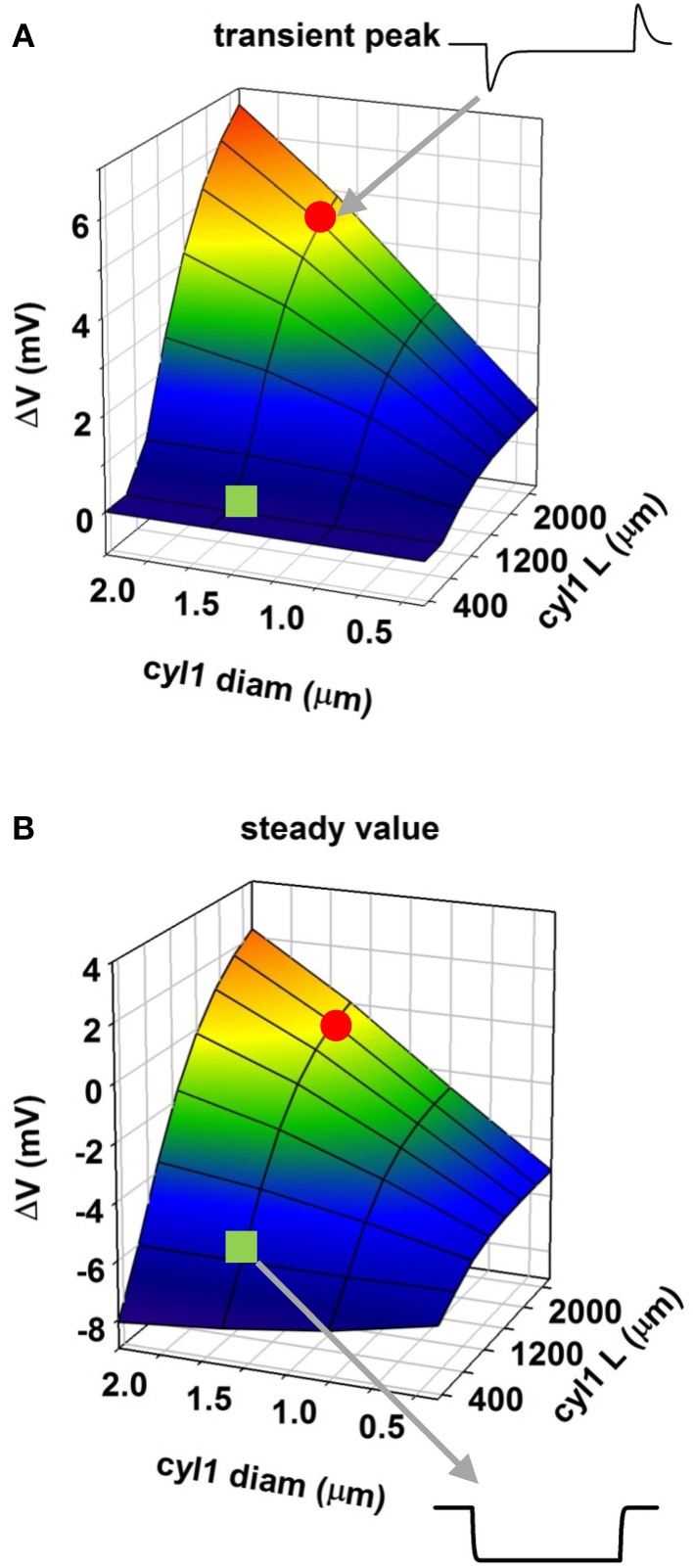
**Modulation of membrane polarization of a passive reduced neuron**. Peak membrane polarization generated by an external field at 565 μm from the origin of a neuron composed of two cylinders (see Figure 1D) as a function of diameter and length of one of the cylinders (cyl1); **(A)** peak polarization during the transient phase; **(B)** steady-state polarization; the red circle and the green square indicate traces in two configurations.

**Figure 3:**
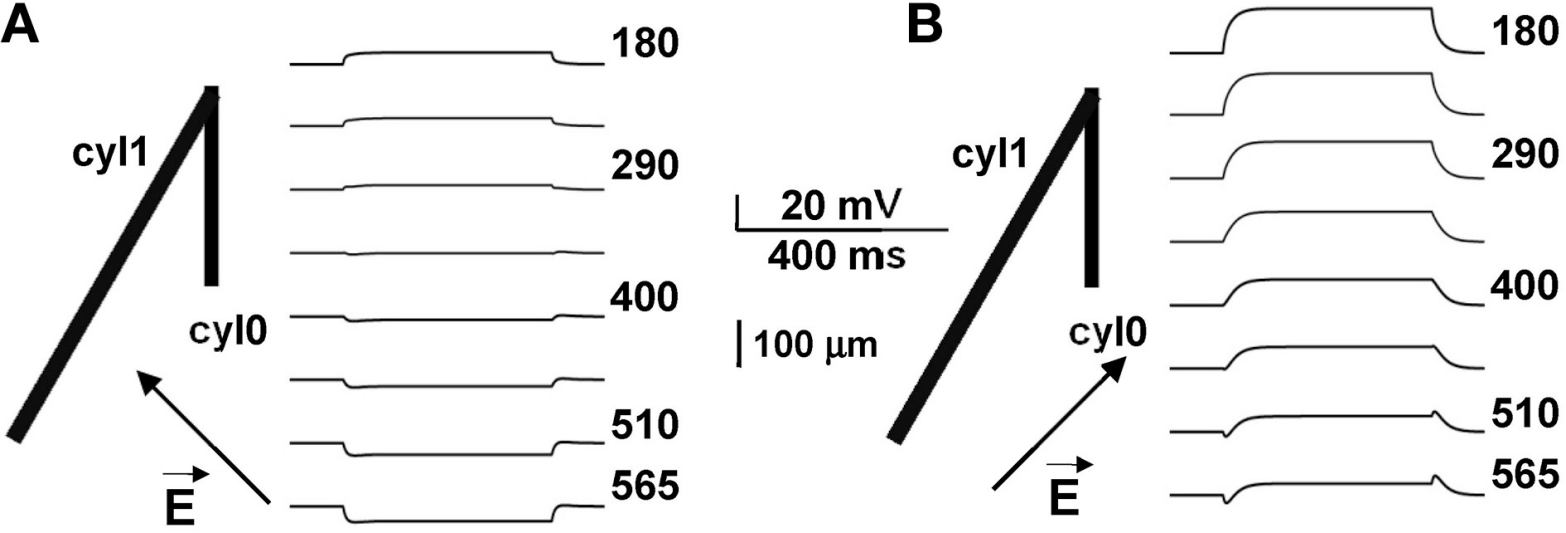
**Biphasic polarization depends on the relative orientation of the field**. Membrane polarization in a neuron composed of two cylinders (as in Figure 1D), for two different orientations of the external field. Arrows indicate field direction (almost orthogonal to cyl1 in **A**, almost parallel in **B**). Biphasic responses can be entirely explained by cell morphology and field orientation, and reflect the differential current flow generated by the field in different branches of the cell.

To evaluate this explanation, we examined the effects of a uniform field on simple cylindrical dendrite models with uniform passive properties (Figures 1B–E). With a single cylinder (cyl0 in Figure 1B, length = 800 μm, and diameter = 1 μm) and a −40 V/m field parallel to its length (φ = θ = 90°) there was no sag in the membrane potential, and the cylinder showed a simple ohmic behavior (Figure 1B). Adding a second cylinder (cyl1, Figure 1C) identical to cyl0 but connected so as to form an angle of 30° with EF did not change the qualitative time course of the membrane potential. However, changing the length and diameter of this second cylinder produced different effects on the time course of membrane potential: a longer branch (3200 μm, Figure 1D) generated a sag, whereas a thicker dendrite (1.5 μm, Figure 1E) changed the steady-state level. Results of a thorough exploration of these effects, using compartment lengths consistent with the average path length of CA1 neurons in rats (Scorcioni et al., 2004), are summarized in Figure 2, where we plot the peak amplitude of the transient (Figure 2A) and the steady-state membrane potential (Figure 2B) relative to resting potential as functions of the length and diameter of cyl1. The response to an EF is also modulated by the orientation of the field with respect to the principal axis of a neuron, as is shown in Figure 3. The two orientations of the field produce significant qualitative differences in membrane depolarization. These results demonstrate clearly that non-uniform membrane resistance is not necessary to produce the experimentally observed biphasic polarization of neuronal membrane potential by an EF field. Instead, biphasic responses can be entirely explained by cell morphology and field orientation, and reflect the differential current flow generated by the field in different branches of the cell.

**Figure 4:**
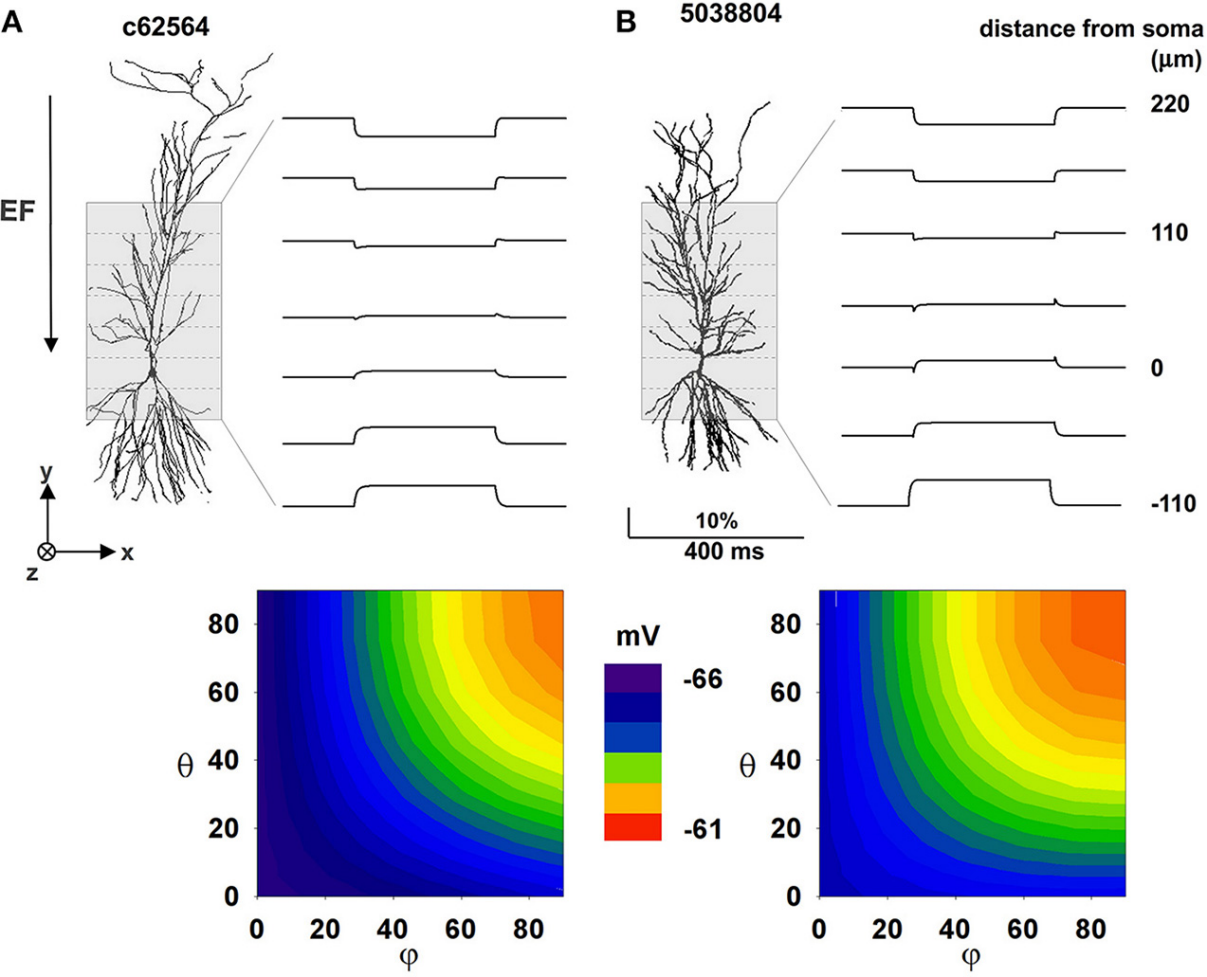
**The effect of a uniform EF is similar in different CA1 passive morphologies**. Average membrane potential during activation of a 40 mV/m EF on two hippocampal pyramidal CA1 neurons with only passive properties. **(A)** Results for cell c62564, **(B)** Results for cell 5038804. Top plots show membrane potential calculated by averaging the membrane potential of all segments within different 55 μm zones along the Y axis (dotted lines); contour plots at the bottom show somatic membrane potential as a function of field orientation. Note that a uniform external electric field interacting with the full dendritic tree of a neuron can generate current flows that cause differential perturbations of membrane potential over the cell surface. Polar coordinates were used in all cases, with θ and φ as the angles with the *z* and *x* axis, respectively.

Our next step was to study the effect of an EF on realistic morphologies with passive properties. In this case, a −40 V/m field was first oriented parallel to the somatodendritic axis. The key phenomena that we needed to reproduce in this case were transients and steady-state values in qualitative agreement with those observed in the experiments; this is important for verifying our models as valid representations of the real system. According to the experimental setup (Figure 10 in Bikson et al., 2004), optical responses from relatively large portions (55 × 180 μ m) of the CA1 region were measured and averaged from a number of slices and trials. Each experimental trace thus contains contributions from a number of membrane segments from different neurons and with different relative orientations with respect to the field. With our single neuron models we cannot aim at a quantitative reproduction, since this would require the implementation of a large population of CA1 neurons and a detailed implementation of the experimental setup. However, we hypothesized that averaging the contribution from all membrane segments within 55 μm zones along the Y axis can give a reasonable approximation of the experimental traces. The results, in terms of the relative depolarization/hyperpolarization from rest are shown in top plots of Figures 4A,B for the two neurons. The traces were qualitatively (but not quantitatively) similar for the two neurons, with large positive or negative polarizations in the distal apical and basal dendrites, and sags corresponding to the on and off times of the field in intermediate regions of the apical dendrites (Figure 4, top plots). Measurements at the soma (Figure 4, bottom plots) suggest that particular field orientations can result in a several mV shift of the membrane potential, with possible consequences for overall neuronal excitability. For both neurons, the maximum effect at the soma was produced by fields oriented along the somatodendritic axis. These results confirm that our models are able to reproduce the main experimental features, and show that a uniform external electric field can interact strongly with the full dendritic tree of a neuron, generating current flows that cause differential perturbations of membrane potential over the cell surface.

**Figure 5:**
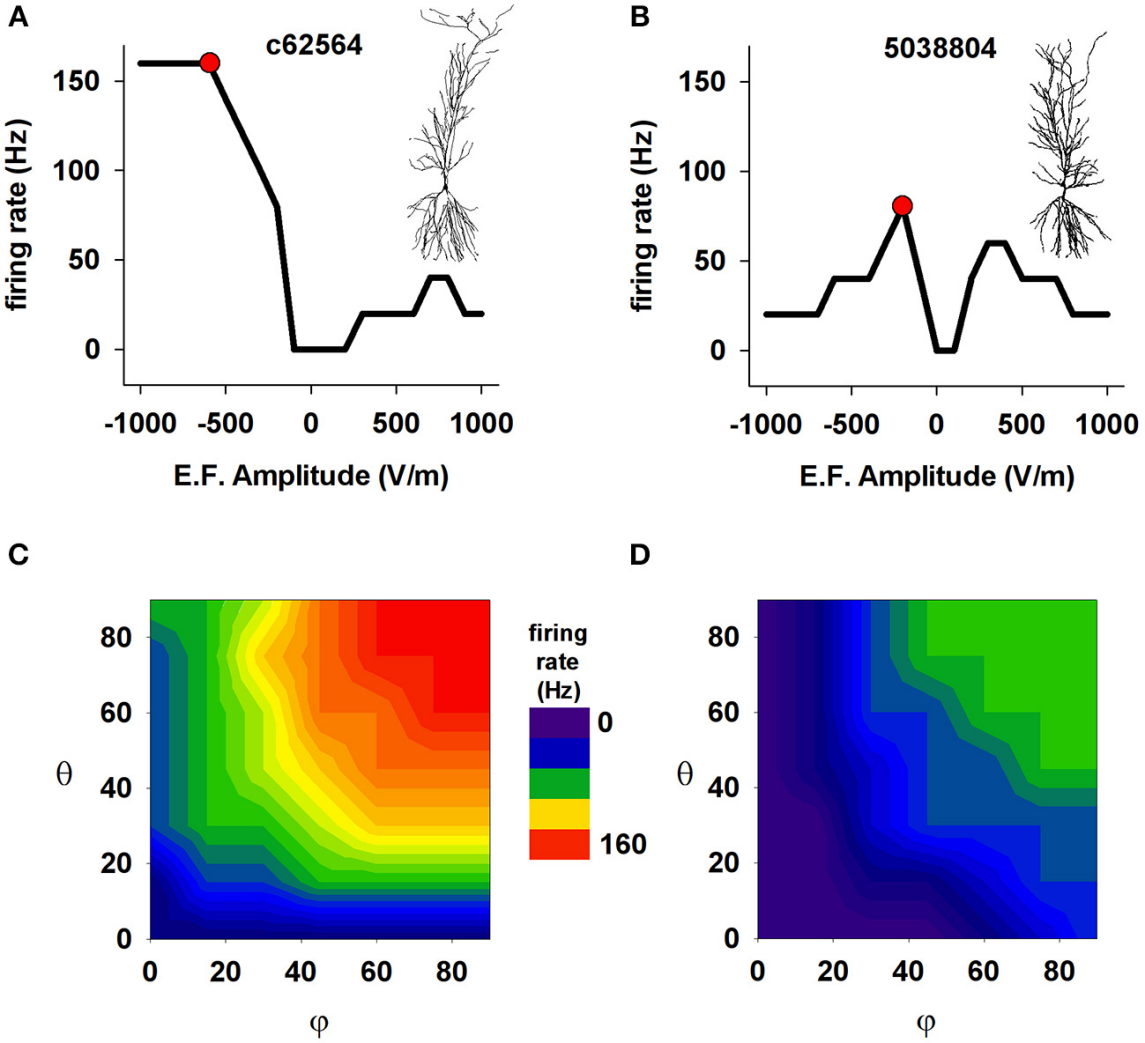
**The effect of a uniform EF is different in different CA1 neurons with active properties**. Number of somatic action potentials generated during a 50 ms exposure to an EF as a function of field amplitude for cell c62564 **(A)** and cell 5038804 **(B)**. For each cell, the red markers indicate the amplitude that elicited the maximum number of spikes. **(C,D)** Number of somatic action potentials elicited in the two neurons during a 50 ms exposure as a function of the field direction relative to the somatodendritic axis. In both cases, the amplitude generating the maximum number of APs was used (red markers in **A,B**). Note that two neurons of the same cell class (hippocampal CA1 pyramidal neurons) can show quite different responses.

These effects, however, can be expected to depend not only on the field strength but also on the specific active properties that neurons express in their dendrites. This is particularly important for pyramidal CA1 cells, which have a rather non-uniform distribution of dendritic channels (reviewed in Migliore and Shepherd, 2002, 2005). This is illustrated in Figure 5, which presents results generated from our anatomically detailed model cells with active membranes (see Methods). Panels A and B show the effect of field strength on the frequency of spikes elicited by exposure to an external (uniform) electric field for 50 ms. For one cell (5038804), high field strengths caused a marked drop in firing rate; this is consistent with experimental findings that very strong fields can depress cell spiking (Bikson et al., 2004), possibly because of depolarization block (Bianchi et al., 2012). The effects of field orientation are shown in Figures 5C,D. Each model cell was subjected to the field strength that generated the most spikes (red markers in the top plots) when aligned with the cell's somatodendritic axis: −500 V/m for c62564, and −250 V/m for c5038804 (red markers in Figures 5A,B, respectively). Taken together with Figures 5A,B, these results reveal that the orientation selectivity of these cells is quite different: one (c62564) has a strong “preferred” field orientation, responding best when the field is aligned with the somatodendritic axis and pointing toward the distal dendrites (i.e., θ = φ = 90°), but the symmetry of the top plot in Figure 5B indicates that the other (5038804) is bidirectional, responding in approximately the same way to fields of opposite sign (i.e., φ = 90° θ = −90°).

The observation that the response of a cell to an EF depends on the cell's anatomical and biophysical properties may be expected. However the fact that two neurons of the same cell class (hippocampal CA1 pyramidal neurons) can show such different responses is surprising. Although the two neurons have the same dendritic distribution of ionic currents, and similar mean path distance in the basal dendrites (220.05 μm for 5038804 vs. 266.32 μm for c62564), their total membrane areas differ (55325.47 μm^2^ for 5038804 and 15493.70 μm^2^ for c62564), as do the average path lengths of their apical dendrites (347.36 μm for cell 5038804 and 455.89 μm for c62564). These results suggest the hypothesis that the path length of the apical dendrites and total cell surface area may be important anatomical factors that modulate EF effects.

**Figure 6:**
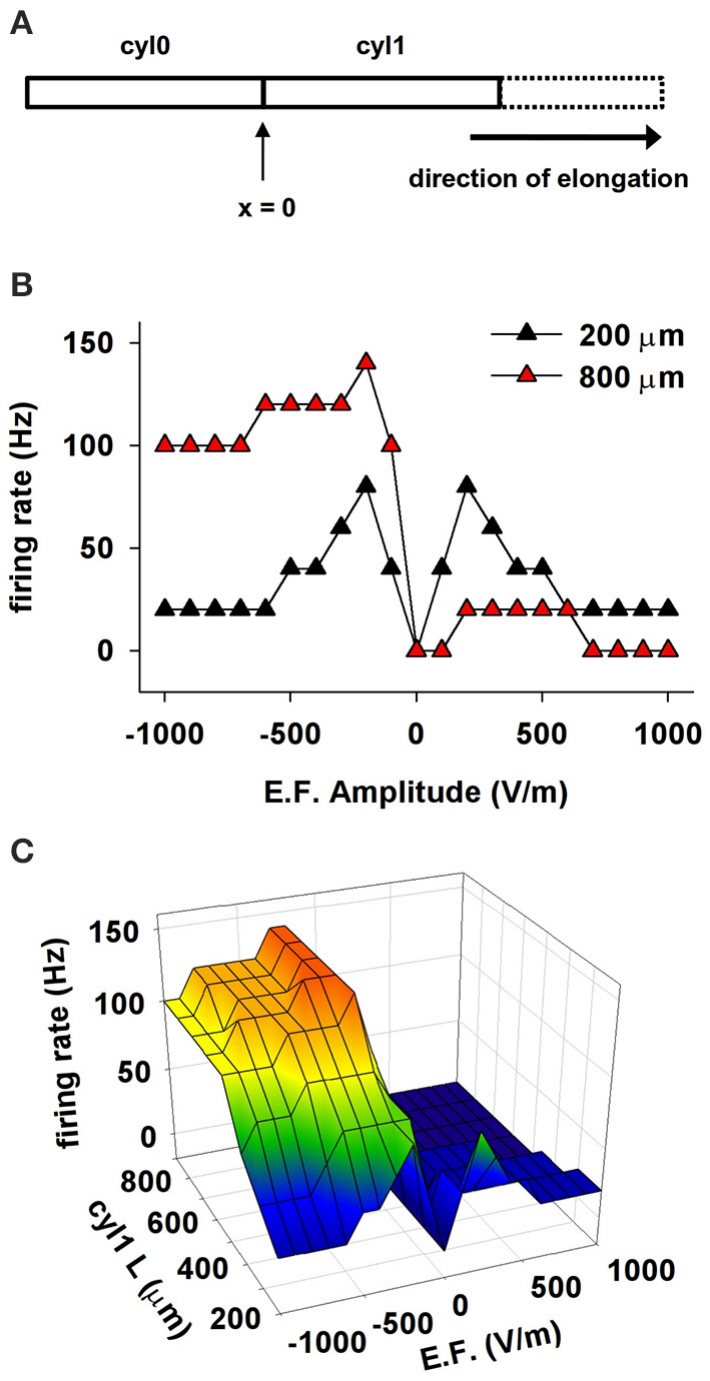
**The effect of a uniform EF on a cell depends on the spatial distribution of active properties**. **(A)** Schematic representation of the cylindrical cell used in these simulations. Membrane passive and active properties were as in CA1 neurons, assuming the soma is at the location indicated as *x* = 0, and cyl1 represents the apical dendrites. **(B)** Number of somatic APs as a function of the EF amplitude and sign using different lengths for cyl1. **(C)** Number of somatic APs generated as a function of cyl1 length and EF amplitude. Note that the spatial extent of the dendritic tree is a major determinant of neuronal susceptibility to an EF.

We tested this hypothesis with a series of simulations that used a cylindrical model composed of two compartments, *cyl0* and *cyl1*, as schematically shown in Figure 6A. Channel densities varied with distance from the soma as in CA1 pyramidal neurons. The independent variables were extracellular field strength (EF) and length of *cyl1*. The results for 50 ms exposures to EFs of different amplitudes are shown in Figure 6B for two representative *cyl1* lengths (200 and 800 μm) and summarized in Figure 6C. Lengthening cyl1 increased the model's orientation selectivity, confirming our hypothesis that the spatial extent of the dendritic tree is a major determinant of neuronal susceptibility to an EF.

**Figure 7:**
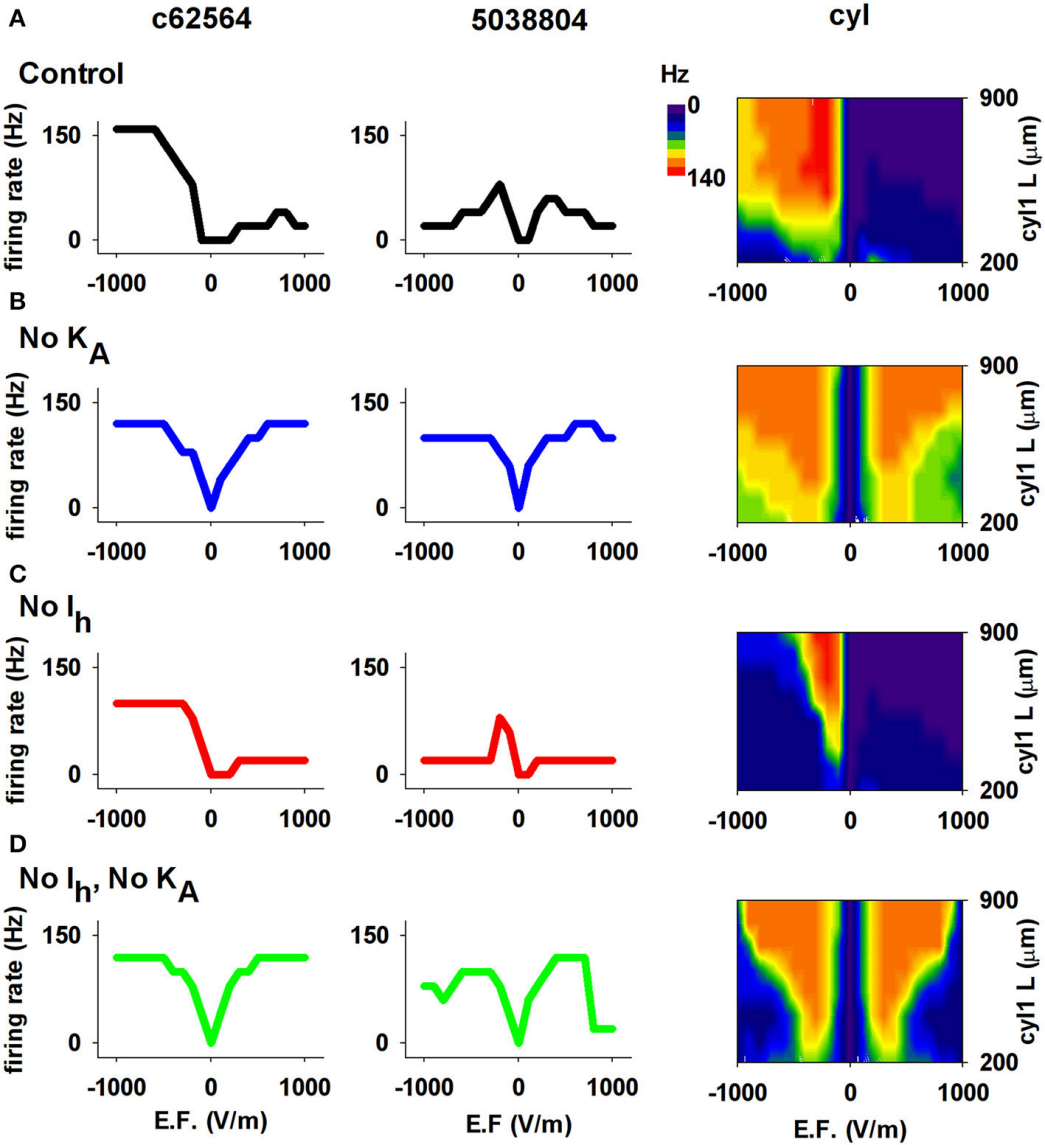
**The I_h_ and K_A_ currents in CA1 neurons determine the effects of an EF**. **(A)** Results using control conditions, i.e., full active properties, for the two realistic morphologies (*left* and *middle*) and the cylindrical model with different lengths (*right*). **(B)** Number of APs elicited by the EF after block of the *K_A_* current; note the overall graphs' symmetry for opposite field polarities. **(C)** Results after block of *I_h_*. **(D)** Results after block of both *I_h_* and *K_A_*. In all cases the field was turned on for 50 ms. Note that both Ih and KA can have significant roles in modulating the response of CA1 pyramidal neurons to external electric fields.

We next investigated the contribution of ionic currents to the sensitivity and directional selectivity of neuronal responses to an EF. Since *I_h_* and *K_A_* expression in CA1 neurons is strongly dependent on distance from the soma, we focused on the effects of these currents. The results are reported in Figure 7 for both 3D reconstructions and for the single cylinder (including the same currents and distribution of the realistic neurons). Under control conditions (Figure 7A), the neuron with the shorter apical dendrites (cell 5038804) and a relatively short cylinder (blue and green region for L≈200 μm in the right plot of Figure 7A) were not selective for a particular field direction, whereas cell c62564 and the longer cylinder showed strong selectivity. Blocking KA (Figure 7B) eliminated the asymmetric response to field direction in all cases, whereas removing Ih (Figure 7C) decreased the cell's sensitivity to the EF but preserved directional selectivity, as demonstrated by the firing rate generated for larger field amplitudes. Taken together, these results indicate that both Ih and KA can have significant roles in modulating the response of CA1 pyramidal neurons to external electric fields.

**Figure 8:**
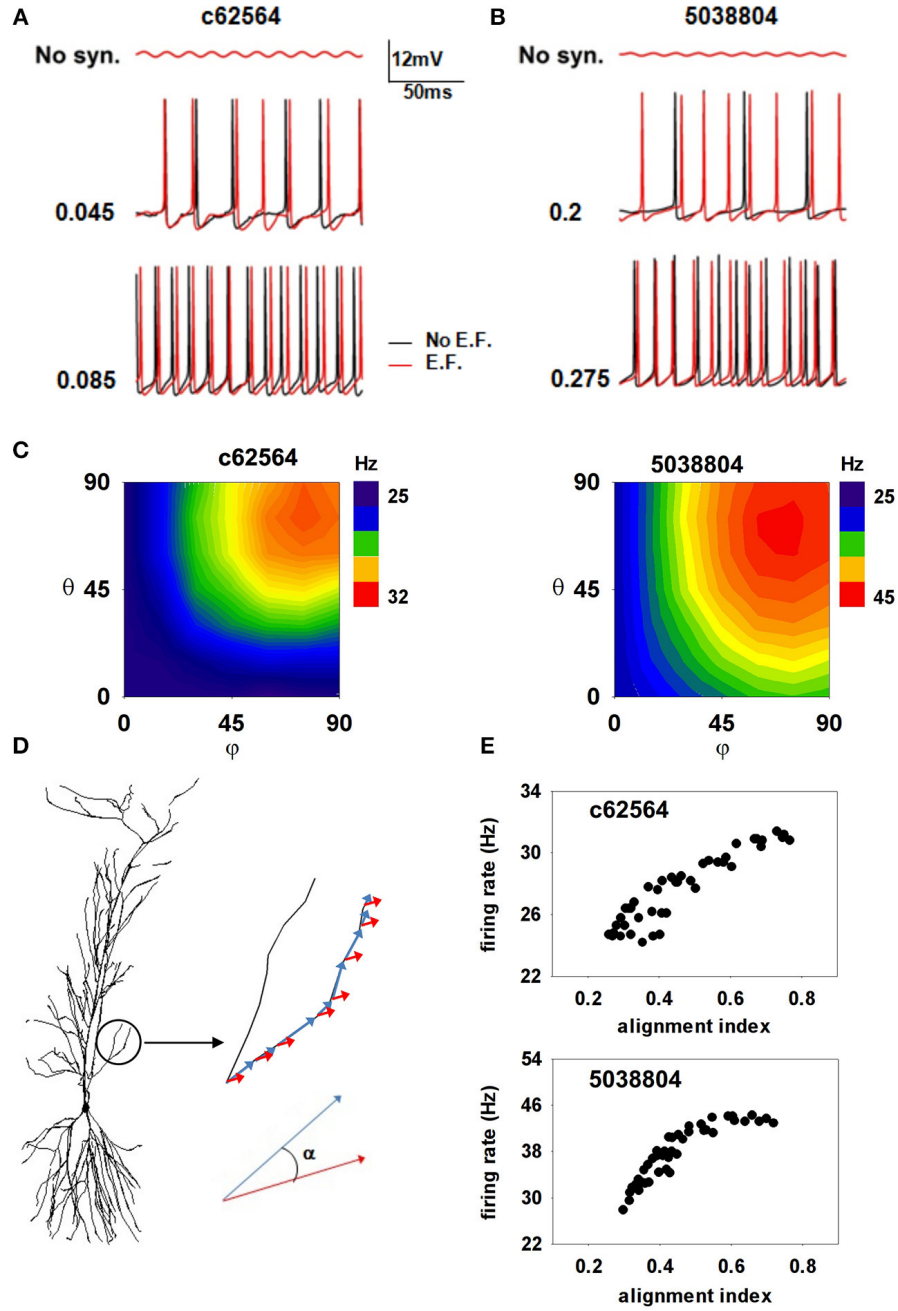
**Oscillating electric field effects on somatic firing depend on alignment of dendrites with the EF**. **(A,B)** Somatic membrane potential of the two cells with (red traces) or without (black traces) a 40 V/m oscillating EF; (top) subthreshold somatic fluctuations in the absence of background activity; (middle) somatic potential in the presence of weak background synaptic activity; (bottom) somatic potential in the presence of strong background synaptic activity; **(C)** Average number of somatic spikes elicited in the two neurons as a function of field orientation in the presence of weak background synaptic activity; **(D)** The alignment of the electric field with the dendritic segments; **(E)** The number of somatic APs generated in the two neurons, as functions of their alignment indices. Note that an external EF, at amplitudes consistent with those measured close to power lines, is able to generate a spurious excitatory activity that greatly depends on the alignment of the dendrites with the field's direction.

We next investigated possible effects of oscillating EFs. It has been suggested that even a subthreshold EF could modulate hippocampal activity, especially in the gamma/theta rhythms range (Ozen et al., 2010; Berzhanskaya et al., 2013), near power line pillars. Anderle et al. (1996) have shown that field amplitudes near these structures can be up to 2520 V/m. Assuming a relative dielectric constant of 67.8 for gray matter (Voigt et al., 2011), we carried out a set of simulations with a 40 V/m field oscillating at 50 Hz (see Materials and Methods). Under these conditions, field-induced oscillations in somatic membrane potential were relatively small (Figure 8, top panels), consistent with those observed in experimental preparations (Deans et al., 2007), and neither neuron exhibited spontaneous activity. To evaluate EF effects in the presence of background activity, we ran simulations (1200 ms long, with the EF turned on from *t* = 200 until the end) with two different levels of random synaptic activity sufficient to elicit spiking at about 24 or 63 Hz (Figure 8 middle and bottom) in the absence of EF. We ran 10 simulations for each of four combinations of conditions (field off/on, firing rate low/high). To highlight the effects on the different morphologies, we chose peak synaptic conductances so as to obtain approximately the same firing rate in both neurons for each activity level. Somatic firing activity was markedly different in the presence of the EF (Figure 8, compare red and black lines in middle and bottom panels), which elicited additional spikes in all cases. For the lower activity level (Figures 8A–B, middle plots), the EF significantly increased the firing frequency of both neurons (to 29.5 ± 1.72 Hz for c62564 and to 41 ± 2.26 Hz for 5038804). With a higher background activity, the firing frequency was not significantly affected by the field. (Figure 8A–B, bottom plots).

Since a small change of dendritic alignment with respect to a time-stationary and spatially oscillating EF can induce a different amount of current in each segment (Anastassiou et al., 2010), we also tested whether the field direction can play any additional role. Plots of the average firing rate for 10 trials, elicited during ten 1 s simulations as a function of the field orientation (Figure 8C) suggest that the field's effect is strongly morphology and direction-dependent. The maximum effect was consistently produced by a field oriented along the somato-dendritic axis (i.e., φ = θ = 90°), consistent with experimental findings (Bikson et al., 2004). To quantify the role of EF orientation, we calculated, for each membrane segment, *i*, an *alignment index* as:

$$alignmentindex=\frac{\sum_{i=1}^{N}L_{i}\left|cos\alpha_{i}\right|}{\sum_{i=1}^{N}L_{i}}$$

where α_*i*_ is the angle between the EF and the *i*-th membrane segment versor, and *L_i_* is the segment length (as illustrated in Figure 8D). High values of the index signify strong overall alignment of the membrane segments with the EF. The firing rate in the presence of a 50 Hz 40 V/m EF, as a function of the alignment index for each EF orientation, is shown in Figure 8E for the two neurons. For both cells the spiking activity induced by the EF tends to increase with the field alignment, with a larger effect for cell 5038804. These results suggest that a subthreshold external electric field, at amplitudes consistent with those measured close to power lines, is able to significantly alter the somatic firing of hippocampal pyramidal neurons, generating a spurious excitatory effect that greatly depends on the alignment of the dendrites with the field's direction.

**Figure 9:**
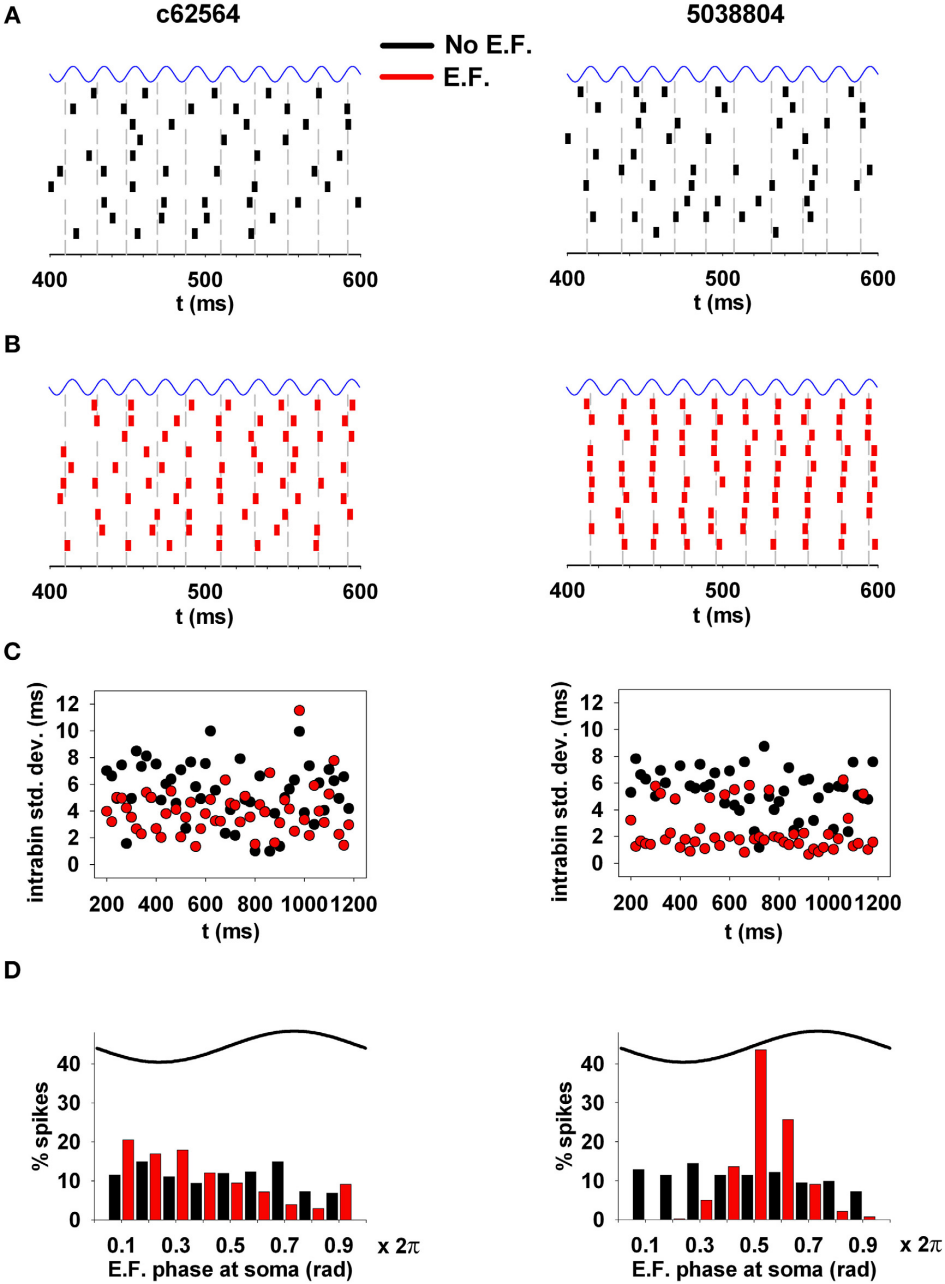
**EFs can alter the synchronization properties of action potentials**. **(A)** *(top)* Spike times in a typical 200 ms time window during 10 trials under control conditions. **(B)** Spike times in the same time window during the same 10 trials in the presence of the electric field. **(C)** Standard deviation of spike times, calculated within individual 20 ms time windows; **(D)** phase relation of the spiking activity with respect to the phase of the underlying oscillation at the soma. Note that oscillating electric fields at a power line frequency can significantly alter the spiking temporal structure of hippocampal CA1 pyramidal neurons. Simulations in **(A,B)** were carried out using weak background synaptic inputs as in Figure 8 (middle); gray lines represent the average spike time calculated from spikes elicited within 20 ms bins.

Next, because of the synchronization effect that subthreshold oscillations can have on neuronal spiking (e.g., Volgushev et al., 1998), we investigated whether an EF oscillating at experimentally observed amplitude and frequency is able to alter the synchronization properties of hippocampal CA1 pyramidal neurons. For this purpose, we analyzed the spike times in 10 trials using weak background synaptic inputs activated at an average frequency of 50 Hz (as in the middle panels of Figures 8A,B), and a 40 V/m field oriented along the somato-dendritic axis (Figures 9A,B). The analysis considered deviations from the average spike time (gray lines in Figures 9A,B) calculated from spikes elicited within 20 ms bins. Due to random synaptic background activity, under control conditions (i.e., without EF, Figure 9A) spike times in different trials should be rather broadly distributed around the average value. Synchronization should manifest as smaller fluctuations from the average value across trials. The raster plots obtained in the presence of the EF (Figure 9B, red plots) show an evident synchronization effect for the two neurons, especially for cell 5038804. For a more quantitative measure of the fluctuations, within each 20 ms time window we calculated the standard deviation of the spike times during the simulations, with respect to the average (Figure 9C). In the presence of the EF (Figure 9C, red points) spike times in both neurons exhibited a significantly smaller deviation from the average spike time, compared to the same simulations without EF (cell c62564, 4 ± 1.8 vs. 5.3 ± 2.3 ms; cell 5038804, 2.4 ± 1.6 ms vs. 4.9 ± 2.2; Wilcoxon Signed Rank test, *p* < 0.002 and *p* < 0.001 for c62564 and 5038804, respectively). Analysis of spiking with respect to the phase of the EF oscillations at the soma, shown in Figure 9D, revealed a significant EF-dependent change (Kolmogorov–Smirnov Test, *p* < 0.001 for both cells). For synaptic inputs activated at 80 Hz (corresponding to the upper frequency limit of the gamma rhythm) there still was a small but significant EF effect for both cells (see Supplementary Figure S2). Taken together these results suggest that oscillating electric fields at power line frequencies can significantly alter the temporal structure of spiking in hippocampal CA1 pyramidal neurons.

**Figure 10:**
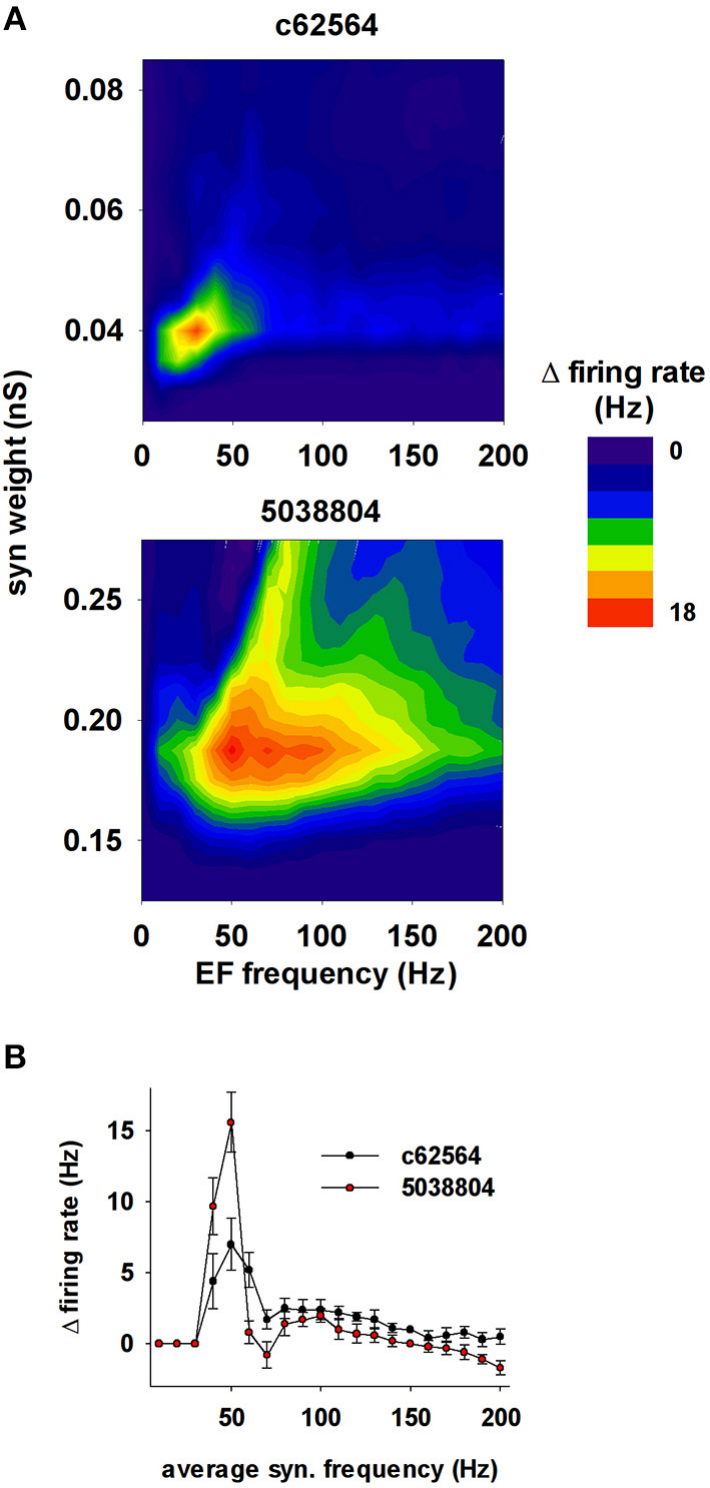
**The effects of the field are greatest around 50 Hz**. **(A)** Change in the firing rate elicited by an external field (with respect to control) as a function of the frequency of the field and the strength of background synaptic activity; (*top*) neuron c62564, (*bottom*) neuron 5038804. **(B)** Change in the firing rate elicited by an external field at 50 Hz as a function of the average activation frequency of synaptic inputs with a peak conductance of 0.04 and 0.2 nS for c62564 and 5038804, respectively.

**Figure 11:**
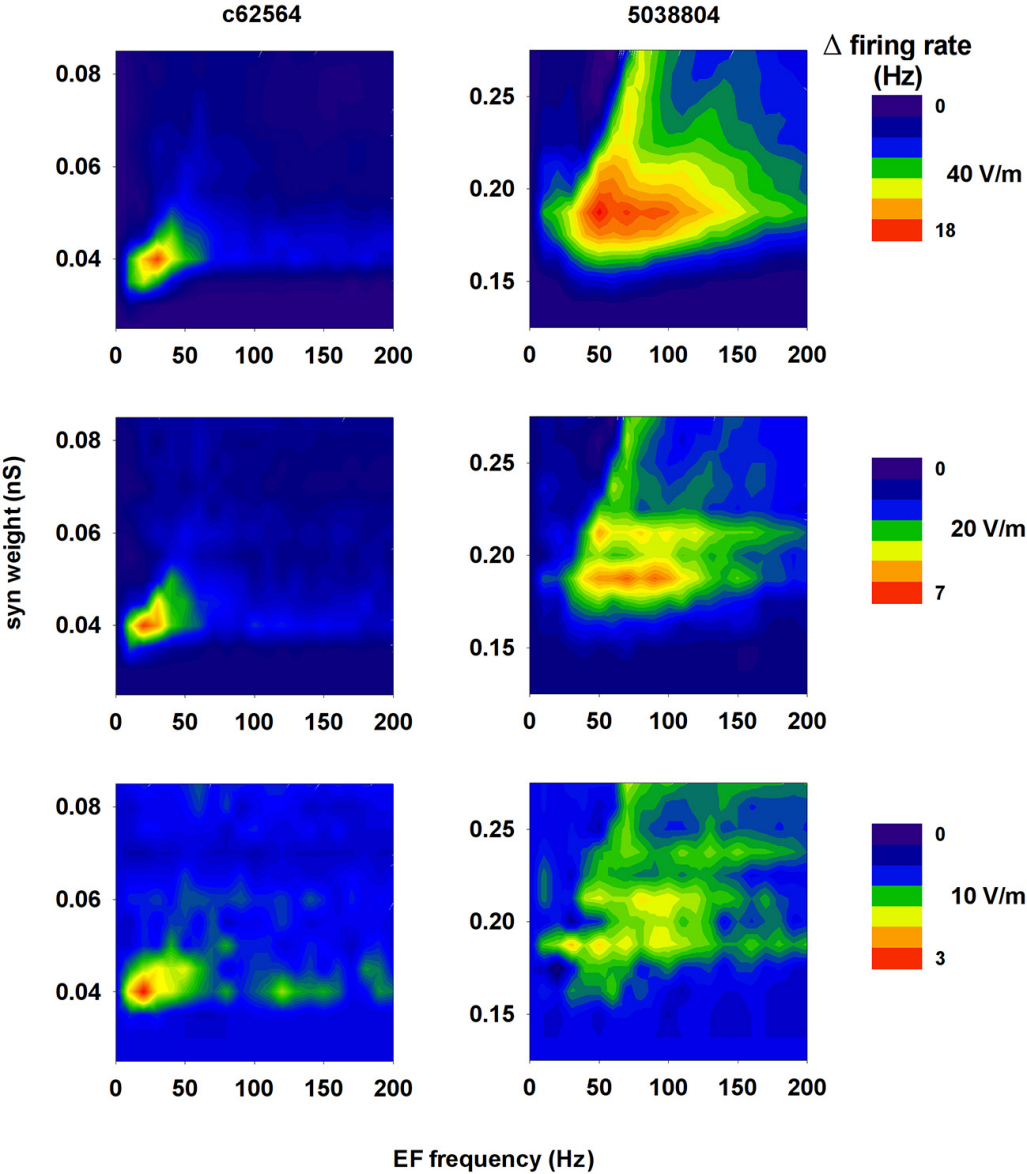
**A 50 Hz field can significantly change the firing rate of CA1 pyramidal neurons within a relatively wide range of field strength**. Change in the firing rate elicited by an external field of 40 V/m (*top*, redrawn from Figure 10A for clarity), 20 V/m (*middle*), and 10 V/m (*bottom*) as a function of the frequency of the field and the strength of background synaptic activity; (*left*) neuron c62564, (*right*) neuron 5038804.

Not all environmental exposure to electrical fields is limited to 50–60 Hz, nor is there any guarantee that synaptic background activity remains constant in the course of daily activities. Therefore we carried out a set of 10 simulations using a 40 V/m EF over frequencies from 0 to 200 Hz in the presence of different levels of background synaptic excitatory activity. The change in firing rate elicited by the field relative to control (i.e., no EF) is plotted in Figure 10A as a function of EF frequency and peak conductance of the synapses generating the random background activity. For cell c62564 the maximum difference from control occurred at an EF frequency of 30 Hz, whereas it was in the 50–60 Hz range for cell 5038804, which is the same used for power lines. Individual cell sensitivity to synaptic background activity was much broader for cell 5038804, which appeared to be sensitive to EFs over wide ranges of EF frequencies and synaptic background activity levels. For an EF at 50 Hz, both neurons were most sensitive to synaptic activation at the same frequency, as shown in Figure 10B for simulations carried out with an average peak synaptic conductance of 0.2 and 0.04 nS for cell c62564 and 5038804, respectively. Qualitatively similar results were obtained for lower values of the EF (10 and 20 V/m, Figure 11), which elicited progressively smaller perturbations in the firing rates of the two neurons. Taken together, these results can be explained in terms of the passive properties of CA1 pyramidal neurons, which happen to have a membrane time constant around 25 ms (Spruston and Johnston, 1992), i.e., in the same range as the EF oscillatory period.

## Discussion

In this work we have highlighted a few mechanisms at the single neuron level that reveal the kind and extent of interactions that may occur between low frequency EFs and hippocampal CA1 pyramidal neurons, which are directly involved in all hippocampus-related cognitive processes. Our results suggest several new experimentally testable predictions. First, the experimentally observed transient biphasic polarization effects on membrane potential can be entirely explained by cell morphology, and are mainly determined by differential current flow generated by the field in membrane segments at different spatial locations. Second, dendritic path length and the distribution of dendritic active channels, such as *I_h_* and *K_A_*, have significant roles in further modulating the differential response of CA1 pyramidal neurons to EFs. Third, in all cases, the overall effects of an EF strongly depend on its spatial alignment with the dendritic segments. Fourth, subthreshold oscillating EFs, at amplitudes measured environmentally, can significantly alter the spike time synchronization properties of a hippocampal CA1 pyramidal neuron. This is important because power line frequencies (50–60 Hz) happen to be in the range of the gamma rhythm, which is deeply involved in cognitive processes, and has a period of the same order of magnitude as the membrane time constant of hippocampal neurons (Spruston and Johnston, 1992). The most obvious predictable consequence of such interactions is a significant perturbation of synaptic plasticity processes that depend on spike timing. This supports the idea that subthreshold low-frequency environmental electromagnetic fields may have significant effects on cognitive processes.

It might be argued that the EF amplitudes used in our simulations (corresponding to 2.5 KV/m in the environment) are observed only in a limited number of hot spots close to power line pillars. Given the rapid decrease of field intensity with distance, this may be considered an unrealistic condition, which cannot be applied to everyday life. However, it has been observed (Leitgeb et al., 2008) that electric emissions from electrical appliances can generate hot spots well above this value. An induction hob or a food processor can generate equivalent electric field strengths above 40 KV/m, much higher than the 5 KV/m recommended by the European Union as the limit of exposure of the general public to low frequency electromagnetic fields (L199/59/EC).

From a modeling point of view, it has been shown that a stationary but non-uniform EF can differentially modulate the spatial distribution of dendritic membrane potential of morphologically detailed passive neurons (Anastassiou et al., 2010). At the network level, a neural mass model implementation of different neuron populations using single point cells, and a phenomenological model for the interaction between an EF and the neuron membrane (Modolo et al., 2013), suggested that power-line fields can affect brain rhythms. Furthermore, using a 2D model network of CA1 pyramidal neurons and interneurons, it has been suggested that subthreshold uniform and stationary EFs can robustly alter the balance between theta and gamma rhythms (Berzhanskaya et al., 2013).

Experimentally, several previous efforts have been carried out to understand the underlying mechanisms and functional consequences of external electric fields. The major problem is that investigations with human subjects (reviewed in Crasson, 2003) have yielded unclear or inconsistent results, with differences between field and control exposure found to be small and difficult to reproduce. Some form of interaction between EFs and cognitive activity has been well established (Beale et al., 1997), and transcranial electric stimulation has been suggested for therapeutic modulation of brain activity (Ozen et al., 2010). However, although the effects of EFs on neurons *in vitro* have been studied and reported in some detail (e.g., Chan and Nicholson, 1986; Bikson et al., 2004), the actual cellular mechanisms and physiological processes that may be involved have been rather difficult to sort out. The main reason for this problem is the large variability and unreliability of experimental observations *in vivo*, especially for working memory tasks (Barth et al., 2010).

In conclusion, the possible cognitive effects of EF generated by power lines are potentially of great public concern. Our findings may explain why behavioral and cognitive effects of EFs have been so difficult to reproduce (Crasson, 2003). Since the overall modulation of the neuronal membrane depends on cellular morphology, ion channel distribution, and relative field orientation, individual neurons in their specific and instantaneous absolute spatial location can interact with EFs in completely different ways. Given the sparse and explicit coding of items in hippocampal neurons (Quiroga et al., 2008), our results predict that the functional consequences of an EF on cognitive processes will be observed only if the (presumably few) neurons directly involved in a specific cognitive task under test are aligned with the field direction.

### Conflict of interest statement

The authors declare that the research was conducted in the absence of any commercial or financial relationships that could be construed as a potential conflict of interest.
