# Supplementary material for: Effects of low frequency electric fields on synaptic integration in hippocampal CA1 pyramidal neurons: implications for power line emissions
Source: Front Cell Neurosci. 2014 Oct 9;8:310. doi: 10.3389/fncel.2014.00310 (PMC4191432; doi:10.3389/fncel.2014.00310)

**Supplementary Fig.S1:**

*(top)* Schematic representation of the external field implementation. The arrow next to E indicates the orientation of the field. The dashed lines indicate the zero potential plane, which is orthogonal to the field and passes through the soma at (0,0,0). Extracellular potential at the dendritic location marked by * is the product of E (field magnitude) and d (orthogonal distance from * to the zero potential plane). The sign of d is positive for points that lie on the cathodal side of the zero potential plane and negative for points on the anodal side of that plane.

*(bottom)* Equivalent circuit of three adjacent compartments along a neurite exposed to an extracellular field. The rings are the boundaries between adjacent compartments. The intrinsic properties of the neurite are depicted as resistors (cytoplasmic resistance between adjacent compartments), capacitors (membrane capacitance), and two terminal "white boxes" that represent each compartment's net transmembrane ionic current. The extracellular field is represented by variable voltage sources that are in series with each compartment's membrane. Because of its spatial position, each compartment, *i*, will be differentially perturbed by field, eliciting a current flow that is responsible for the overall effects observed at the soma.

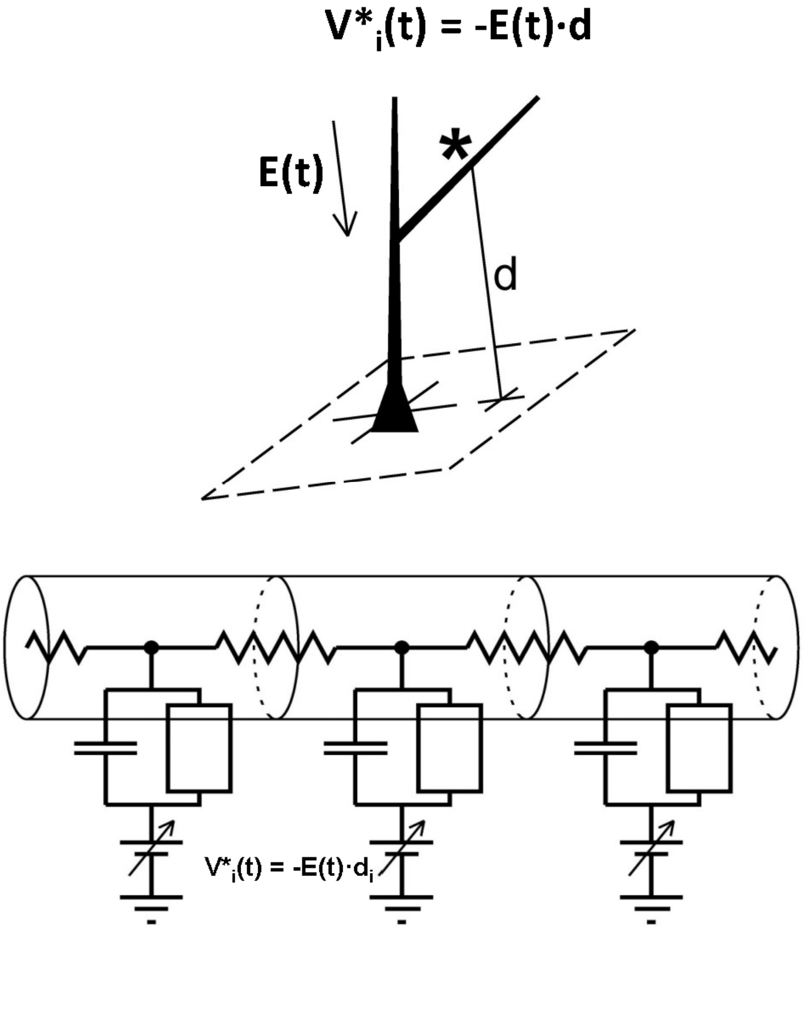

Supplement: Supplementary file 1 [file DataSheet1.PDF]
