# Supplementary material for: Effects of low frequency electric fields on synaptic integration in hippocampal CA1 pyramidal neurons: implications for power line emissions
Source: Front Cell Neurosci. 2014 Oct 9;8:310. doi: 10.3389/fncel.2014.00310 (PMC4191432; doi:10.3389/fncel.2014.00310)

Supplementary Fig.S2:

Phase relation of the spiking activity with respect to the phase of the underlying oscillation at the soma, for a background synaptic activity at 80 Hz and peak synaptic conductance of 0.04 nS (cell c62564) and 0.2 nS (cell 5038804); for both neurons there was a statistically significant difference between results under control and EF (Kolmororov-Smirnov test, p <1e-4 for both cells).

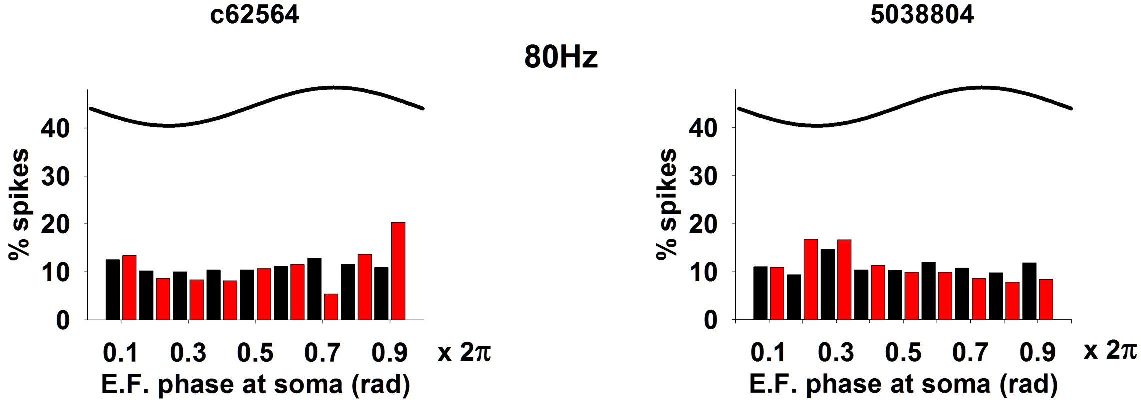

Supplement: Supplementary file 2 [file DataSheet2.PDF]
